# Canine Systemic Insecticides Fluralaner and Lotilaner Induce Acute Mortality of *Triatoma gerstaeckeri*, North American Vector of the Chagas Disease Parasite

**DOI:** 10.4269/ajtmh.23-0300

**Published:** 2023-09-25

**Authors:** Rachel E. Busselman, Italo B. Zecca, Gabriel L. Hamer, Sarah A. Hamer

**Affiliations:** ^1^ Department of Veterinary Integrative Biosciences, Texas A&M University, College Station, Texas;; ^2^ Department of Entomology, Texas A&M University, College Station, Texas

## Abstract

Chagas disease is a health concern for humans and animals across the Americas, and control options targeting the triatomine vectors of *Trypanosoma cruzi*, the causative agent of Chagas disease, are limited. Host-targeted interventions may be a useful and underused tool in controlling the spread of *T. cruzi* from vectors to hosts. Domestic dogs are known to be key bloodmeal hosts for triatomines as well as *T. cruzi* reservoirs and may be an effective and practical target for host-targeted insecticide deployment. We hypothesized that treating dogs with commercially available systemic insecticides (labeled for flea and tick control) would result in mortality of triatomines after consuming treated blood. We enrolled 16 privately owned dogs into five treatment groups to receive either fluralaner (Bravecto) or lotilaner (Credelio), alone or in combination with ivermectin. Blood from dogs before the initiation of treatment served as controls. Blood was collected 0, 7, 30, 45, and 90 days after the initial canine insecticide treatment and fed to 10 *Triatoma gerstaeckeri* nymphs through a membrane feeder, and survival was tracked daily for 7 days and weekly thereafter. All triatomines in the control and ivermectin groups survived the initial period, with no significant difference in long-term survival. In contrast, 99.7% of triatomines that fed on blood from dogs treated with either fluralaner or lotilaner died within 3 days. Although the impact of canine treatment on suppressing vector populations is unknown, fluralaner and lotilaner appear to be a compelling option for an integrated vector management approach to triatomine control.

## INTRODUCTION

Chagas disease is a neglected tropical disease endemic to Latin America and the southern United States. Caused by the protozoan parasite *Trypanosoma cruzi*, Chagas disease can manifest as cardiac damage and gastrointestinal disease in humans, dogs, and other mammals.^1^^–^^5^ One of the primary pathways of *T. cruzi* transmission occurs when feces from an infected triatomine enters a mammalian host, primarily through a bite site or ingestion of the infected insect or its feces.^1^^,^^6^ As such, established triatomine vector populations are necessary for endemic transmission of *T. cruzi*.

In endemic countries in Latin America, such as Argentina, Venezuela, and Brazil, successful vector control interventions to interrupt transmission of *T. cruzi* have been ongoing for decades.^7^^–^^9^ In these settings where domestic triatomine populations predominate, intensive insecticide treatment of homes has been effective to reduce human Chagas disease cases.^7^^,^^9^^–^^11^ Multicountry initiatives such as the Southern Cone Initiative and Initiative of the Countries of Central America for Control of Vector-Borne and Transfusional Transmission and Medical Care for Chagas Disease (IPCA) have played key roles in reducing the incidence of human Chagas disease cases in domestic transmission cycles.^9^^,^^12^^–^^14^

Although residual spraying campaigns are effective at reducing *T. cruzi* infections and domestic populations of triatomines, the peridomestic and sylvatic populations of triatomines still pose a challenge for vector management. Triatomines progress through multiple nymphal instar stages before molting into adults, which is the only life stage that can fly to disperse; otherwise, triatomines of all life stages disperse by walking. Triatomines living in nearby peridomestic foci can lead to persistence of triatomine populations even after insecticide spraying, and in areas where triatomine species are primarily peridomestic or sylvatic, residual spraying has limitations on the success of controlling triatomine populations.^9^^,^^15^^,^^16^ For example, in endemic regions of the United States, indoor spraying is less likely to be effective because local triatomine species are primarily encountered in peridomestic or sylvatic environments and nymphs are less commonly found in homes.^17^^,^^18^ *Triatoma gerstaeckeri*, a peridomestic species of triatomine associated with burrows and wildlife nests, is the most commonly encountered species in Texas and occurs in parts of New Mexico and 10 states of northern Mexico.^19^^–^^23^ No insecticides are labeled for or are widely used against triatomines in the United States, limiting targeted vector management approaches to prevent *T. cruzi* transmission. Recommendations currently include turning off exterior lights at night, reducing triatomine habitat around kennels and homes by clearing woody debris, and applying structural pest insecticides around areas where dogs are housed.^24^ Development of alternative complementary insecticide interventions are necessary.

Host-targeted insecticides may be a complementary method of triatomine control through a process known as xenointoxication, in which a host animal is treated with a systemic insecticide, creating toxic bloodmeals for the target vector. Various topical treatments have had mixed results; for example, fipronil applied to dogs induced slight to no mortality in triatomines,^25^ imidacloprid applied to pigeons induced triatomine mortality at higher doses,^26^ and pour-on cypermethrin applied to goats and chickens induced high triatomine mortality shortly after application.^27^^,^^28^ Ivermectin, which is a common heartworm prevention and antiparasitic endectocide, when given to dogs and chickens, can acutely induce triatomine mortality (within 1–2 days of treatment) but has a quickly waning efficacy, resulting in no triatomine mortality just 3 days after chicken treatment.^29^^–^^31^

A newer class of systemic insecticide, isoxazolines, shows promise for effective and applicable triatomine control in addition to the flea and tick prevention for which it is marketed. Studies have shown fluralaner (Bravecto) and afoxolaner (NexGard) induce nearly 100% mortality in *T. infestans* within 5 days up to 51 days post-dose.^32^ Fluralaner has also been shown to induce 100% mortality in *T. infestans* within 24 hours regardless of permethrin susceptibility, and mortality within 4 days of feeding on a treated host remained high (> 47%) up to 120 days post-treatment.^33^ Additionally, when *T. brasiliensis* were exposed to dogs treated with fluralaner, they experienced 100% mortality up to 7 months after a single treatment of dogs with fluralaner, and declining levels of triatomine mortality lasted through 11 months.^34^ Further, fluralaner has been deployed in an endemic setting of the Argentine Chaco region, where prior control measures have resulted in pyrethroid-resistant infestations of *T. infestans*.^35^^,^^36^ In homes where fluralaner was deployed on dogs, the population of pyrethroid-resistant triatomines was significantly reduced and almost all households remained infestation-free up to 10 months after canine fluralaner treatment.^35^ Over a 22-month period of treating dogs with fluralaner in a community, household infestations of triatomines dropped significantly, and the prevalence of *T. cruzi* infection in the vectors decreased, reducing the risk of *T. cruzi* infections in humans in these homes.^36^

Dogs are a promising target host for systemic insecticide treatments in many areas where Chagas disease is endemic. Dogs are reservoir hosts for *T. cruzi*, common bloodmeal sources for triatomines, and often in close contact with humans, making them integral to *T. cruzi* transmission dynamics and potential targets for control efforts.^37^^,^^38^ Dogs are also known to be a source of *T. cruzi* in domestic transmission settings.^39^ A serosurvey of dogs from an area in Arequipa, Peru, that was previously sprayed with insecticide found 12.3% of dogs were positive for *T. cruzi*, and the authors suggest dogs may serve as sentinels for disease reemergence.^40^ Additionally, modeling studies of domestic transmission cycles in Latin America have shown that the presence of dogs inside a home changes the bloodmeal composition of domestic triatomine populations, and removing domestic dogs from households would significantly reduce the risk of *T. cruzi* infections to humans.^41^^,^^42^

In the southern United States, canine infections are common, although a lack of standardized reporting masks the true veterinary burden.^43^^,^^44^ Across animal shelters in diverse ecoregions of Texas, canine Chagas disease was just as common as heartworm disease, with 18.1% of shelter dogs infected.^45^ A survey of shelter dogs in Louisiana revealed at least 6.9% were seropositive, and similarly 6.4% of dogs were positive in a Tennessee serosurvey.^46^^,^^47^ Additionally, more than 57% of dogs were infected in kennel environments in central Texas,^48^ and nearly a third of uninfected dogs developed a new infection over the course of 12 months in multidog kennels with known peridomestic triatomine populations.^49^ In a recent synthesis of bloodmeal data from triatomines in the United States, nearly 23% of all bloodmeals identified and 30% of *T. gerstaeckeri* bloodmeals were from canines (*Canis lupus* and *Canis* spp.), further emphasizing their role in maintaining triatomine populations in the United States.^5^ Using dogs as a target for vector control may have the additional benefit of helping to address *T. cruzi* infection and subsequent Chagas disease in dogs, which is a veterinary concern.^3^^,^^50^

In this study, we test the efficacy of two commercially available systemic insecticides (fluralaner, Bravecto^®^; lotilaner, Credelio^®^) alone and in conjunction with ivermectin (an endectocide commonly prescribed for heartworm prevention) on the blood feeding success and survival of *T. gerstaeckeri*. We test individual systemic insecticides as well as in combination with ivermectin because these preventatives are often used together by dog owners to prevent fleas, ticks, mites, heartworms, and other internal parasites. Finding safe and effective vector control methods may aid in reducing both human and canine exposure to *T. cruzi* and incidence of Chagas disease. Host-targeted interventions may be a useful tool to improve the integrated vector management approaches available for disease prevention.

## MATERIALS AND METHODS

### Canine enrollment.

Privately owned, apparently healthy dogs of any breed or sex that were at least 6 months of age were enrolled over the course of 6 months (May–August, 2020) through a veterinary clinic in Omaha, Nebraska. Dog owners were required to disclose the history and type of systemic insecticides used previously, if applicable. Dogs were enrolled through this clinic because many dogs in this northern latitude may not receive heartworm/flea/tick prevention in the winter months based on their owner’s preference, which allowed for the collection of pretreatment blood samples in early spring as a control group. The control group comprised the subset of dogs that had no history of prior insecticide use or for which insecticides were applied at least 4 months prior; after the pretreatment blood draw, these control dogs were immediately crossed over into a treatment group. A total of 16 dogs were enrolled into one of five treatment groups: ivermectin only, fluralaner only, fluralaner+ivermectin, lotilaner only, and lotilaner+ivermectin. Dogs with a history of regular or recent prevention were placed into the treatment group to match or add to their prior or existing treatment. For example, a dog who had regularly received fluralaner but no ivermectin may have been placed in the fluralaner only or the fluralaner+ivermectin treatment group, whereas a dog that had received no flea/tick prevention and no ivermectin may have been placed in any treatment group.

**Figure 1.**
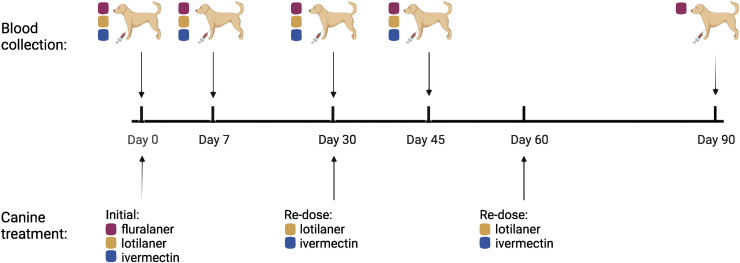
A timeline of treatment and blood collection protocol throughout the study. Fluralaner is given once every 90 days, and lotilaner and ivermectin are given every 30 days. Groups given only lotilaner and/or ivermectin were sampled up to day 45, whereas groups given fluralaner were sampled through day 90. In all cases, treatments were given after blood was drawn. Created with BioRender.com.

At the visit of enrollment (day 0), initial blood samples were collected and the first dose of the systemic insecticide was administered by the owner either at the veterinary clinic or later that day at home. Both fluralaner and lotilaner were given to the dogs enrolled in this study at the labeled dose to kill fleas and ticks (by mouth every 12 weeks or 30 days, respectively), and ivermectin was given at the labeled dose to kill heartworms and other parasites (by mouth every 30 days). Samples at day 0 from dogs with no history of either an endectocide or systemic insecticide comprised the control group. Day 0 samples from dogs with a history of systemic insecticides and/or ivermectin use were excluded from analyses. Blood samples of 7 to 9 mL were collected on days 0, 7 (range 5–14 days), 30 (range 27–42 days), and 45 (range 44–63 days) after the initial treatment of all groups, and an additional day 90 sample (range 87–96 days) was obtained for dogs receiving fluralaner (Figure 1). The blood collection timeline was based on the marketed activity period of each product for its on-label uses of preventing flea and tick infestations (fluralaner and lotilaner) and heartworm prevention (ivermectin). Both lotilaner and ivermectin have 30-day activity periods and were redosed every 30 days within our 3-month study, whereas fluralaner has a 90-day activity period and was therefore given only once. For all treatments, day 7 samples were considered an early measure of efficacy in the treatment’s activity period. For lotilaner and ivermectin, the day 30 sample represents the label endpoint, and the day 45 sample serves as a midpoint in between two doses of continuous treatment. For fluralaner, the day 45 sample serves as a midpoint assessment, and the day 90 sample represents the label endpoint.

Blood samples were placed in a lithium heparin tube (BD Vacutainer^®^, VWR International, LLC, Radnor, PA) and sent overnight to the laboratory for insect trials.^51^

### Insect trials and monitoring.

All triatomines (*T. gerstaeckeri)* used in this study were maintained in the U.S. Department of Agriculture Animal and Plant Health Inspection Service Plant Protection and Quarantine–approved BSL2 quarantine triatomine colony on Texas A&M University’s campus. In the colony before the trials, triatomines were fed a maintenance diet of defibrinated rabbit blood (HemoStat Laboratories, Dixon, CA) weekly using Hemotek feeders (Hemotek Ltd., Lancashire, United Kingdom), as previously described.^52^

Ten third, fourth, or fifth instar *T. gerstaeckeri* nymphs from colony-reared triatomines were used in each trial. The colony originated from adults captured in Texas between 2016 and 2019, with all progeny used for the experiments between the F1 and F3 generation. Access to maintenance feeding was restricted before the trial (duration of triatomine starvation was 2–48 days, average 19 days, *n =* 600) to increase the affinity to feed on dog blood during the trial.

Trials were conducted in a bioBUBBLE BSL-2 and ACL-2 containment enclosure (bioBubble Inc, Fort Collins, CO). Groups of 10 triatomines were contained in plastic Nalgene containers with filter paper (Whatman Filter Paper, Sigma-Aldrich Inc., Darmstadt, Germany) lining the bottom of the tub and placed vertically to allow them to crawl to a mesh secured over the top to allow feeding. At least 3 to 24 hours before the initiation of a trial, all insects were individually marked with a unique color nail polish on the caudal aspect of the dorsal abdomen to facilitate individual identification and tracking. For the surviving insects subjected to long-term analysis, fresh markings were applied after molting (discussed subsequently).

Individual glass membrane feeders were attached to a warm water bath, which was set to 40°C, and a water pump using a series of short plastic tubes. Each feeder was covered with Parafilm (Parafilm M All-Purpose Laboratory Film, Amcor PLC, Neenah, WI) and individually placed over the mesh of a triatomine container to allow the nymphs to feed. Each group of 10 triatomines was offered 5 to 6 mL of blood over a 2-hour period. The glass membrane feeders were cleaned and disinfected with 10% bleach for 24 hours, then thoroughly rinsed with water before reuse.

The amount of blood triatomines consumed during a trial was scored in two ways: visual engorgement scores and pre- and posttrial weights. Engorgement was subjectively scored into four categories: not fed, little fed, medium fed, and engorged.^53^^,^^54^ Weights were measured on a VWR P-series/C balance (VWR International, LLC). A scale calibration error occurred during a single trial that impacted the pretrial triatomine weights and was corrected before the posttrial weights were obtained; data were salvaged by applying a correction to pretrial weights that was allowed under the assumption that unfed bugs gained no weight. Both scoring methods were used in tandem to identify each triatomine as “fed” or “unfed” for analyses. The maximum scale variation we observed for individuals identified as “not fed” (0.01 g) served as the weight cutoff for a triatomine to be considered fed.

After the trial, triatomines were monitored every 24 hours for 7 days. Survival (alive, dead, moribund) was noted for each bug daily. Moribund triatomines—or triatomines that appeared to be almost dead—were labeled “dead” in our analyses because no bugs went on to recover after becoming “moribund.” After the initial 7-day monitoring period, surviving triatomines were placed on a colony maintenance diet with blood offered every other week and monitored weekly for survival for 89 weeks after the initial trial. When triatomines used in the trials molted during the posttrial observation period, they were remarked with the same color of nail polish; nymphs were again marked on the caudal aspect of the dorsal abdomen, whereas adults were marked on the dorsal aspect of the pronotum.

Additional data on surviving triatomines (molting success and fecundity) are presented in the Supplemental materials.

### Statistical analysis.

We used a multinomial linear regression and a generalized linear model (using a gaussian distribution) to test whether blood feeding success was affected by the treatment group, life stage, number of days the triatomines were starved, or the number of days after initial treatment. These analyses included all 600 triatomines in the trials. All other analyses were conducted with only those 515 triatomines that were considered to have fed during a trial.

To assess what variables affected whether a bug was alive or dead 7 days posttrial, a generalized linear mixed effect model was used, including only those bugs that fed during the trial. This model included the treatment groups, day of blood collection after insecticide treatment (0, 7, 30, 45, 90), life stage at start of trial, and percent weight change as fixed effects, and dog ID and room temperature as random effects.

The difference in long-term survival was investigated using a Kaplan–Meier survival analysis and Cox regression model. Only those bugs that were in the ivermectin and control groups that fed during the trial were used as these were the only bugs to survive the acute time frame. For 13 triatomines, a death date was not recorded; thus, these triatomines were considered lost to follow up, and the last date the triatomine was known to be alive was included as a censored data point in our analyses. All data manipulation and statistical analyses were conducted in Microsoft Excel^55^ and Program R^56^ (with R Studio^57^) using the packages nnet,^58^ lme4,^59^ and survival.^60^^,^^61^

## RESULTS

### Enrolled dogs.

A total of 16 client-owned dogs were enrolled in this study: four male and 12 female. Six dogs that either had no history of prior systemic insecticide use (*n =* 3) or prior insecticide use 131 days to 3 years prior (*n =* 3) provided day 0 blood that served as a control group; these dogs were then enrolled into a treatment group. Of the five treatment groups, four had three dogs each enrolled (lotilaner, fluralaner, lotilaner+ivermectin, and ivermectin) and one (fluralaner+ivermectin) had four dogs enrolled. One dog in the lotilaner group was lost to follow-up after the 30-day visit.

At the start of the study, enrolled dogs weighed between 5.4 and 41.8 kg (21.3 kg average) and ranged in age from 1.5 to 13 years (6.75 years average). Breeds enrolled in the study included Labrador Retriever (*n =* 3), Queensland Blue Heeler Mix (*n =* 1), German Shepherd (*n =* 2), Miniature Dachshund (*n =* 2), Poodle (*n =* 1), Goldendoodle (*n =* 3), Shipoo (*n =* 1), Peekapoo (*n =* 1), Boxer mix (*n =* 1), and mixed breed (*n =* 1).

### Triatomine feeding success.

A total of 600* T. gerstaeckeri* nymphs were used across 60 trials. Trials included 40 third, 171 fourth, and 388 fifth instar triatomines (one nymph was unable to be identified to life stage). Because of the relatively small sample size, third and fourth instars were grouped together for analyses. Of the 600 triatomines used in the trials, 515 (85.8%) were categorized as fed at the end of their trial. For the 85 unfed insects, 49 nymphs (8.2%) apparently decreased in weight (weight changes ranged from −0.0001 to −0.0059 g; average: −0.001 g), and 36 insects known to have not fed had an increased weight up to 0.0100 g (average 0.0018 g), likely reflecting measurement error.

**Figure 2.**
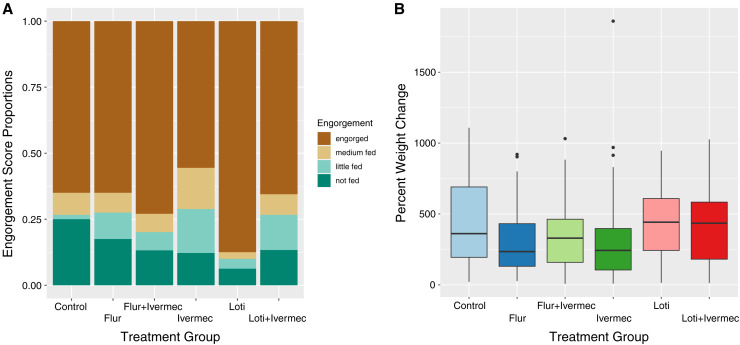
Triatomine feeding success across treatment groups. (**A**) Qualitative engorgement scores show the proportion of bugs in each engorgement category for each treatment group, including all 600 bugs in our analyses. (**B**) Quantitative measures of the percent weight change of insects during a feeding trial in each treatment group including only those 515 insects that were considered fed. Flur = fluralaner; Ivermec = ivermectin; Loti = lotilaner.

Considering all 600 triatomines in the trials (Figure 2A), there were no differences between triatomines that fed and those that did not across treatment groups, life stages, days starved, and days post–initial treatment (multinomial linear regression: *P* = 0.17–0.87, 95% CI: −1.50 to 2.78). There were more triatomines categorized as “little fed” in every treatment group compared with the control group (multinomial linear regression: *Z* scores = 20.8–41.6, *P* < 0.001, 95% CI: −1.50 to 2.78) and fewer “medium fed” and “little fed” bugs in fourth instars than fifth instars (*Z* = −3.10 and −3.69, *P* = 0.001 and < 0.001, 95% CI: −2.08 to −0.47 and −2.24 to −0.68). Nymphs that were scored as “little fed” had significantly fewer days since the canine insecticide treatment than control bugs (*Z* = −3.03, *P* = 0.002, 95% CI: −0.04 to −0.01).

**Figure 3.**
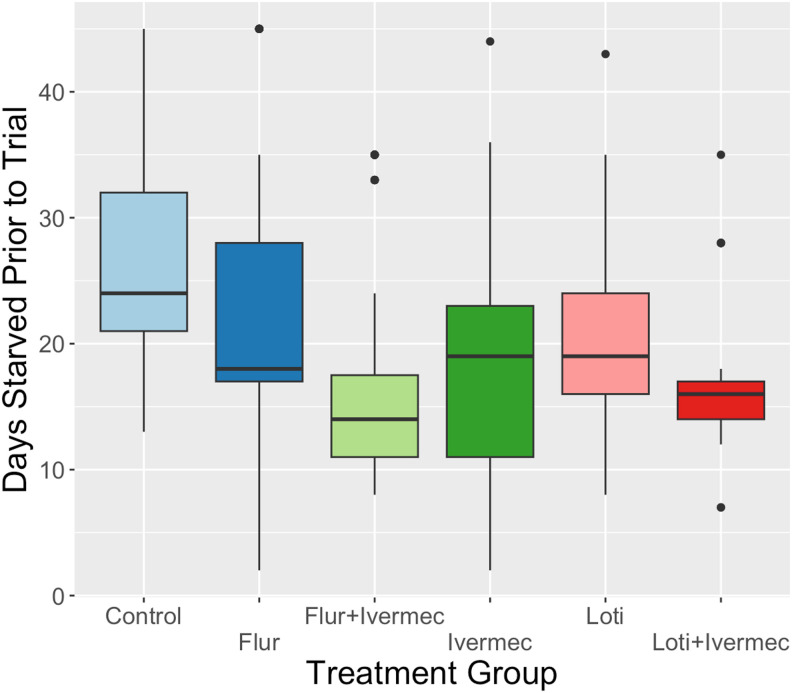
The quantitative measures of the days a triatomine was starved prior to the trial, including only the 515 insects that fed during the trial. Flur = fluralaner; Ivermec = ivermectin; Loti = lotilaner.

When considering only those triatomines that fed (*n =* 515, Figure 2B), fluralaner had significantly smaller percent weight changes than the control (general linear model [GLM]: *t* value = −2.426, *P* = 0.0156, 95% CI: −226.46 to −24.06), and ivermectin had a marginally smaller percent weight change compared with the control (GLM: *t* value = −110.859, *P* = 0.060, 95% CI: −226.23 to 4.506). The third and fourth instars (grouped together for analyses) had a significantly higher percent weight change when compared with fifth instars, increasing weight gain by 403.9% on average during a feeding, versus the average 339.3% increase in weight by fifth instar nymphs (GLM: *t* value = 78.027, *P* < 0.001, 95% CI: 32.62–123.44). The number of days a triatomine was starved before the trial was associated with a slight increase in the percent weight change (Figure 3, GLM: estimate = 2.65, *t* value = 1.927, *P* = 0.054, 95% CI: −0.045 to 5.35). There were no differences in the days after the dogs’ treatment in triatomine percent weight change. Converting the weight change of triatomines to the volume of blood consumed (using a 1 g:1 mL ratio) in those that fed, triatomines consumed between 10.6 μL and 1,029 μL of blood, with the average triatomine consuming 361 μL during one feeding.

### Acute mortality.

**Figure 4.**
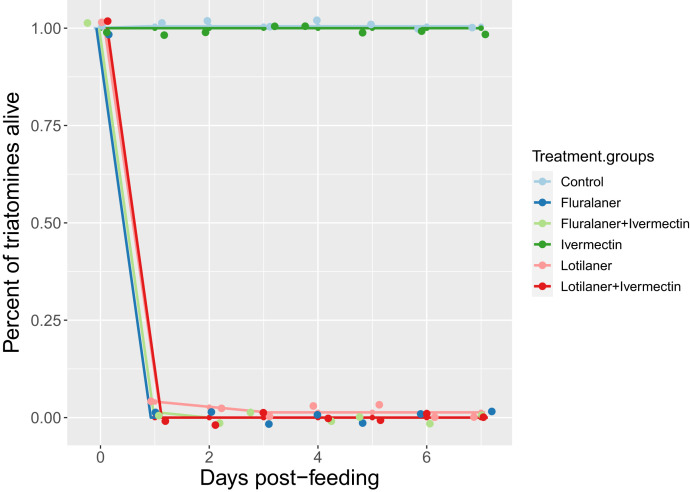
The percent of fed triatomines (*n =* 515) that were alive every 24 hours over the first 7 days after initial trial separated by treatment group. Data from triatomines fed on dogs at different days after initial treatment (7, 30, 45, 90 days) and across life stages are combined given lack of differences.

No vectors in the ivermectin or control groups died during the first 7 days after the trial (Table 1). Using a generalized linear mixed model (GLMM), the treatment group significantly predicted triatomine survival up to 7 days post-feeding. Triatomines that fed on fluralaner, fluralaner+ivermectin, lotilaner, or lotilaner+ivermectin died acutely when compared with control or ivermectin (GLMM: *t* values = 81.4 to 92.2, *P* < 0.001, 95% CI: 0.96–1.02) (Figure 4, Table 1). Across all groups, the days a dog’s blood sample was collected post-treatment (days 7, 30, 45, 90) and life stage of triatomine nymphs at the start of the trial had no significance to whether the bug died in 7 days.

**Table 1:** Summary of triatomine mortality across treatment groups, days after the initial canine treatment, and engorgement categories

Treatment group	No. fed/total	“Not fed” mortality	No. fed that died within 7 days	“Little fed” mortality	“Medium fed” mortality	“Engorged” mortality
Control	45/60	1/15	0/45 (0%)	0/1	0/5	0/39
Ivermectin	79/90	0/11	0/79 (0%)	0/15	0/14	0/50
7	28/30	0/2	0/28 (0%)	0/3	0/5	0/20
30	29/30	0/1	0/29 (0%)	0/9	0/6	0/14
45	22/30	0/8	0/22 (0%)	0/3	0/3	0/16
Fluralaner	99/120	5/21	99/99 (100%)	12/12	9/9	78/78
7	26/30	2/4	26/26 (100%)	2/2	2/2	22/22
30	26/30	2/4	26/26 (100%)	5/5	2/2	19/19
45	22/30	0/8	22/22 (100%)	5/5	5/5	12/12
90	25/30	1/5	25/25 (100%)	–	–	25/25
Fluralaner + Ivermectin	139/160	3/21	139/139 (100%)	11/11	11/11	117/117
7	34/40	1/6	34/34 (100%)	9/9	3/3	22/22
30	37/40	1/3	37/37 (100%)	1/1	5/5	31/31
45	33/40	0/7	33/33 (100%)			33/33
90	35/40	1/5	35/35 (100%)	1/1	3/3	31/31
Lotilaner	75/80	2/5	74/75 (98.7%)	2/3	2/2	70/70
7	27/30	1/3	27/27 (100%)	1/1	2/2	24/24
30	29/30	0/1	29/29 (100%)	–	–	29/29
45	19/20	1/1	19/19 (100%)	2/2		17/17
Lotilaner + Ivermectin	78/90	2/12	78/78 (100%)	12/12	7/7	59/59
7	23/30	2/7	23/23 (100%)	6/6	–	17/17
30	28/30	0/2	28/28 (100%)	1/1	4/4	23/23
45	27/30	0/3	27/27 (100%)	5/5	3/3	19/19

### Comparing long-term survival in insects that survived the first 7 days.

**Figure 5.**
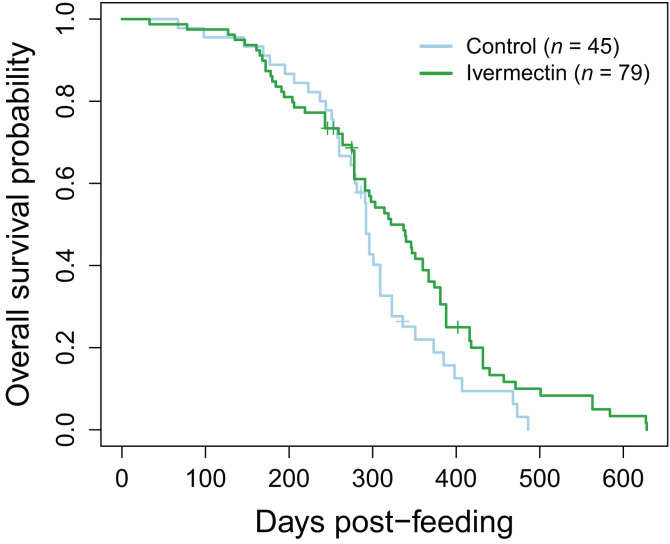
Kaplan–Meier curve comparing the survival of *Triatoma gerstaeckeri* nymphs that ingested blood from dogs treated with ivermectin versus insects that ingested untreated control blood. Insects in all other treatment groups (fluralaner, fluralaner+ivermectin, lotilaner, lotilaner+ivermectin) died within 3 days of the trial and were not included in these analyses. Vertical tick marks indicate vectors that were censored at the last date they were known to be alive when the death date was unknown.

We assessed long-term survival differences of triatomines after they had been returned to maintenance colony feeding conditions; this was restricted to insects in the control and ivermectin-only treatment group because all other insects died acutely. In total, 75% (45/60) of control and 88% (79/90) of ivermectin triatomines fed during their trials. Survival data were collected up to 89 weeks (628 days) after the initial trial. When comparing long-term survival in bugs that fed during the trials, there was no difference between the two groups in time it took the triatomines to die (Cox regression model: *P* = 0.073; 95% CI: 0.46–1.03; hazard ratio = 0.69; Figure 5). The level of engorgement during a feeding had no effect on how long it took triatomines to die (Cox regression model: *P* > 0.087; 95% CI: 0.62–2.76; hazard ratios = 1.04–1.61). Molting and fecundity traits of insects from long-term monitoring are included in the Supplemental Materials.

## DISCUSSION

We tested the efficacy of fluralaner (Bravecto^®^), lotilaner (Credelio^®^), and ivermectin on the blood feeding success and survival of *T. gerstaeckeri* nymphs. We found that both fluralaner and lotilaner induced near-complete mortality in *T. gerstaeckeri* nymphs within 24 hours of ingestion, whereas ivermectin did not affect triatomine mortality acutely or over time.

The observed mortality effect for all treatment groups was consistent across the recommended dosing period of each prevention, up to the 30-day period for lotilaner and the 90-day period from the dogs given fluralaner. Ivermectin given alone did not induce mortality in triatomines, and it did not alter the efficacy of fluralaner or lotilaner to induce rapid triatomine mortality. The 100% mortality due to fluralaner is consistent with results from similar studies. Canine fluralaner treatment induced 100% mortality in *Rhodnius prolixus* adults within 48 hours after live canine feeding trials, and they observed 100% mortality up to 20 weeks after the initial canine treatment.^62^ This extends the length of effective treatment past our assessment of up to the recommended 12-week dosing interval. Further, fluralaner has been shown to induce 100% mortality in *Triatoma brasiliensis* nymphs within 5 days of feeding up to 7 months after the initial canine treatment, decreasing the frequency of required insecticide deployment compared with monthly or more frequent insecticide applications.^34^ These researchers also showed that triatomine mortality reaches 0% efficacy only 12 months after the initial canine fluralaner treatment.^34^ In contrast, another study found that mortality in *T. infestans* nymphs dropped to 70% to 81% by 90 days after canine treatment with fluralaner, and by 210 days, nearly all *T. infestans* nymphs that fed on treated blood remained alive.^33^ Our study adds to the growing body of evidence that fluralaner can induce mortality in triatomines, including *T. gerstaeckeri*, at least through the recommended dosing interval and at the currently marketed dose. It also provides novel evidence for the triatomine lethality effects of lotilaner.

*Triatoma gerstaeckeri* nymphs were used in this study because of the species’ important role in the ecology and epidemiology of *T. cruzi* transmission in the southern United States and northern Mexico. It is one of the most widely geographically dispersed triatomine species in Texas and was the most common triatomine species found in and collected by humans in a multistate community science program.^19^^,^^22^^,^^63^^–^^65^ Additionally, *T. gerstaeckeri* are known to feed on dogs and are commonly encountered in and around areas that house dogs.^5^^,^^48^^,^^66^^,^^67^ Adult *T. gerstaeckeri* have previously been characterized by high *T. cruzi* infection prevalences in natural peridomestic and sylvatic settings, often exceeding 50%, with some studies reporting up to 100% infection prevalence in *T. gerstaeckeri* collected around dog kennels.^48^^,^^63^^,^^67^^,^^68^ As key vectors of *T. cruzi* in peridomestic transmission cycles with regular contact with dogs and other peridomestic hosts,^5^ the 100% mortality of *T. gerstaeckeri* in this study highlights the potential of targeting peridomestic triatomine species through host-targeted insecticides.

To conduct the feeding assays in this study, we used membrane feeders, which are an accessible way to feed triatomines without having to maintain or introduce live animals to a laboratory environment.^69^ Overall, triatomines consumed between 10 and 1,029 μL of blood during a feeding, with the average blood intake of 361 μL. The volume of blood intake is much more than the 2 to 130 μL reported to have been consumed by *Rhodnius ecuadoriensis* when feeding on anesthetized live Swiss mice.^70^ Although it is possible membrane feeding artificially increases the bloodmeal size for triatomines, we observed mortality in bugs that had only fed on 10 μL of treated blood, and we found no association of the amount of blood consumed to when a triatomine died. Thus, it is likely the mortality effects would hold in a natural setting where triatomines were receiving bloodmeals from a live host, where additional factors such as host behavior may limit the amount a kissing bug consumes.^71^

Using membrane feeders, we found triatomines that had been starved longer were more likely to consume larger volumes of blood during the trial. We also found third and fourth instars (grouped for analyses) were more likely to become engorged when compared with fifth instar nymphs. Additionally, statistically, triatomines scored as “little fed” were more likely to have been in a trial with fewer days since canine insecticide treatment. However, we believe this to be an artifact of small sample sizes with only one triatomine being little fed at both the day 0 and day 90 timepoints. Triatomines feeding on dogs treated with fluralaner consumed less blood than other treatment groups. This is in contrast to another study that showed triatomines feeding directly on fluralaner-treated dogs were more likely to take a complete bloodmeal.^32^ However, our results support the conclusion of this and another study that show fluralaner does not affect the success of blood feeding.^32^^,^^33^ Regardless of differences in the percent weight changes across groups, almost all triatomines that fed on fluralaner or lotilaner died acutely, even when individuals in that group consumed slightly smaller volumes.

When compared with fluralaner and lotilaner at the time points we tested, oral ivermectin did not result in triatomine mortality. A prior study showed that ivermectin, when given to dogs dosed with subcutaneous injections (Detomax^®^) at 20 mg a.i./kg, induced 83.3% mortality in *T. infestans* within 35 days of the trial in bugs fed within 24 hours of the injection.^29^ Triatomine mortality declined after the first 24 hours after the dog’s treatment, and the authors concluded that the metabolism of ivermectin within the first 48 hours results in ivermectin concentrations too low in the system to affect triatomines thereafter. Giving dogs subcutaneous injections of ivermectin results in a slower absorption time (32–36 hours versus 4 hours) but similar plasma concentrations as orally administered ivermectin.^72^ Of note, TriHeart^®^, the ivermectin treatment used in this study, was given at the recommended oral heartworm prevention dose of 0.006 mg/kg.^72^ Because our first sampling timepoint was at 7 days after initial treatment, we do not expect to see a mortality effect on triatomines because the ivermectin was likely already metabolized and at undetectable limits before our blood sampling. Further, of the surviving insects subjected to long-term monitoring, we did not detect a difference in time-until-death for the insects that fed on ivermectin versus control blood, further supporting that the drug was not at a level that impacted the insects.

A limitation of the current study is that we did not quantify active ingredients in dog plasma, which would have aided in comparing the dose of the active ingredient in the product given to dogs that occurred in the plasma during the indirect feeding by triatomines.^30^^,^^73^ Based on the lack of mortality of triatomines feeding on the blood from dogs treated with ivermectin, the level of ivermectin was likely below detection limit by mass spectrometry. Given our observed high mortality of triatomines feeding on the blood of dogs treated with fluralaner or lotilaner at 90 days after initial treatment, future studies should extend the range of treatment paired with measuring the active ingredient in plasma to help identify the necessary dose and frequency of dog treatment to sustain triatomine control.

Finding safe and effective vector control methods is a much-needed avenue of research to reduce the risk of Chagas disease in humans and animals. We found both fluralaner and lotilaner cause acute mortality in *T. gerstaeckeri* nymphs throughout the duration of the labeled dosing interval, indicating they may be effective tools in integrated pest management approaches to control triatomine populations in areas where triatomines are feeding on dogs. In settings with domestic triatomine infestations, host-targeted insecticides may serve as a complementary insecticide management approach to residual spraying. For example, giving resident dogs one dose of fluralaner has drastically reduced the triatomine abundance and household infestations in the Argentine Chaco, and *T. cruzi* vector infections and the frequency of humans as bloodmeal sources also were reduced in these communities.^35^^,^^36^ The potential length of efficacy of fluralaner is up to 7 months, and thus it is an excellent candidate for intervention efforts as well because it does not have to be readministered monthly.^34^

In settings with primarily peridomestic or sylvatic cycles of *T. cruzi* transmission, host-targeted insecticides may fill a gap in the otherwise sparse toolbox of effective triatomine management. In areas where dogs often serve as bloodmeals, or if systemic insecticides can be extended to include treatment of wild vertebrate hosts of triatomines, it is possible fluralaner or lotilaner deployment may have a measurable effect of triatomine population sizes or *T. cruzi* infection prevalences. For example, in the Lyme disease system, fluralaner baits designed for *Peromyscus* mice were deployed in the field and successfully reduced the number of tick larvae found on mice.^74^ Also, in the West Nile virus system where birds play a major role in transmission, chickens and wild Eurasian collared doves were fed bird feed treated with ivermectin to kill feeding mosquitoes, effectively killing more *Culex* mosquitoes that fed on birds with detectable ivermectin in their plasma.^75^ Additionally, a topical formulation of fluralaner is commercially available for cats, and fluralaner has been used in a variety of nontarget animal species, including the American black bear (*Ursus americanus*) and the bare-nosed wombat (*Vombatus ursinus*), among others.^76^^–^^78^

Ivermectin and fluralaner have also been tested in chickens—a different key peridomestic host species—on their ability to kill triatomines, finding fluralaner killed kissing bugs and ivermectin did not, similar to our study.^30^ Although oral and injectable ivermectin at these 30-day doses do not provide lasting insecticide effects against triatomines, long-term veterinary formulations of ivermectin may be worth investigating for their potential cost-effective application in endemic areas.^79^ Recent mathematical models of peridomestic transmission of *T. cruzi* show using host-targeted insecticides in environments where dogs are key triatomine hosts may reduce triatomine populations over time, especially in high transmission environments, but the changes to the risk of *T. cruzi* to the dogs are not clear.^80^^,^^81^

Canine systemic insecticides, including fluralaner and lotilaner, show promise as a “One Health” intervention to kill multiple vector species simultaneously (e.g., triatomines, fleas, ticks, mosquitoes). If these effects scale in the field to achieve vector population suppression, then both animal and human health may benefit from reduced vector contact.

## Supplemental Materials


Supplemental materials
