# Supplementary material for: Canine Systemic Insecticides Fluralaner and Lotilaner Induce Acute Mortality of Triatoma gerstaeckeri, North American Vector of the Chagas Disease Parasite
Source: Am J Trop Med Hyg. 2023 Sep 25;109(5):1012–21. doi: 10.4269/ajtmh.23-0300 (PMC10622482; doi:10.4269/ajtmh.23-0300)
Supplement: Supplementary file 1 [file tpmd230300.SD1.pdf]

Supplemental File: Long-term monitoring of triatomines fed control vs ivermectin treated dog blood: molting and fecundity traits

Statistical methods:

Molt analyses: Molt analysis was applied only to insects that fed during the trials (as determined based on individual nail polish identification); insects that neglected to feed (did not gain weight) were excluded. Molting success was analyzed using a chi-squared test of independence to compare molting success between groups (triatomines fed control dog blood vs. ivermectin dog blood). Kaplan Meier analysis was performed to investigate the differences between groups in days between the trial and first molt; triatomines that did not molt after the trial were excluded from the analysis. There was a single triatomine that did not molt before death and the date of death was unknown; this insect was excluded in the analysis. A Cox regression model was used to quantify the Kaplan Meier days-to-molt analysis.

Fecundity: As triatomines in the control and ivermectin groups molted into adults, we monitored the number of females and the number of eggs that were laid as well as the hatching success of those eggs. Both fed and unfed bugs from the trials were housed together and eggs laid cannot be attributed to fed vs. unfed insects. The number of weeks each group's females were producing eggs varied, such that the control bugs were monitored for 39 weeks, while the three groups of ivermectin bugs (originating from trials of 7, 30, and 45-days post initial dog treatment) were monitored 31, 8, and 30 weeks, respectively. A repeated measures ANOVA was used to determine if there were differences between control and ivermectin groups over time in the number of eggs laid per female per day.

Results:

Molt analysis: We found that significantly more triatomines in the ivermectin group molted before death (68/79, 86%) compared to the control group (25/44, 57%; p-value = <0.001). There was no difference in the days from trial to the first molt between ivermectin and control bugs (p-value = 0.859; 95% CI = 0.60-1.53; HR = 0.96; Figure S1).

Fecundity: The number of females in each group were tracked every week (Figure S2a). These females were monitored for up to 39 weeks, and the number of eggs that were laid was 1176 (control group) and 3502 (ivermectin groups combined; Figure S2b). Data were normalized to the number of eggs/female/day and no difference were noted between control vs. ivermectin

triatomines (p-value = 0.111, Figure S3). Out of the 1176 eggs that were laid in the control group, 712 hatched (60.5%), and out of the 3502 eggs laid in the three ivermectin groups, 1476 hatched (42.1%) with no consistent trends in proportion hatched over time (Figure S4).

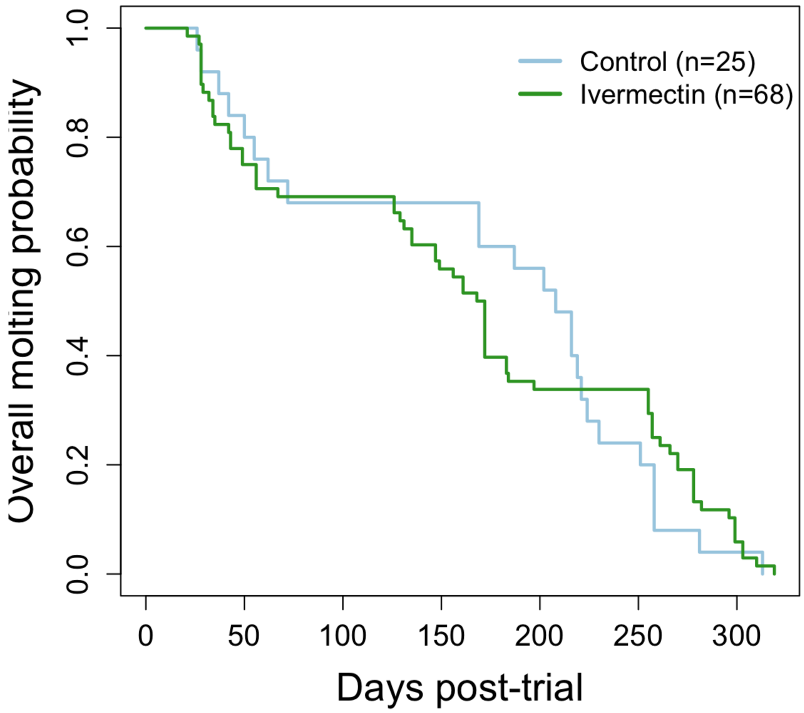

Figure S1: Kaplan Meier curve comparing the days it took to the first molt after the trial in *Triatoma gerstaeckeri* nymphs that ingested blood from dogs treated with ivermectin versus insects that ingested untreated control blood. This graph excludes all bugs from the control and ivermectin groups that did not molt after the trial.

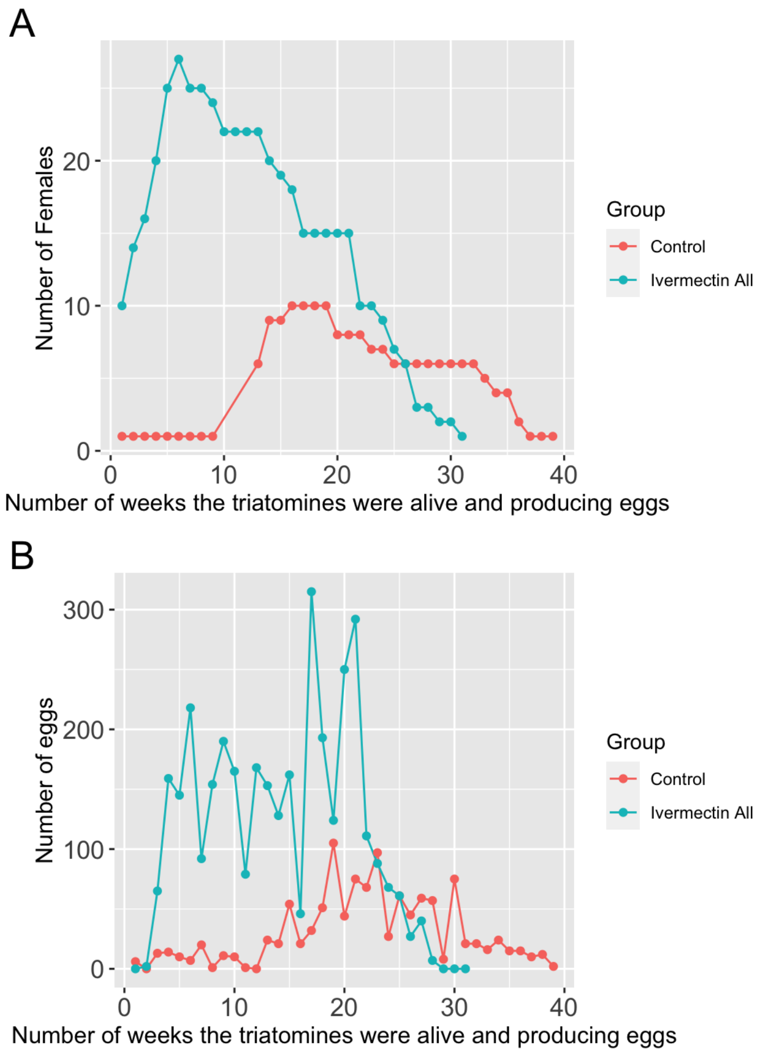

Figure S2: Raw numbers of (A) Females and (B) Eggs per group (Control vs Ivermectin; data were combined from days 7, 30, and 45) over time. 'Week 1' was the first instance either a nymph molted to a female (A) or a female produced eggs (B) in each group.

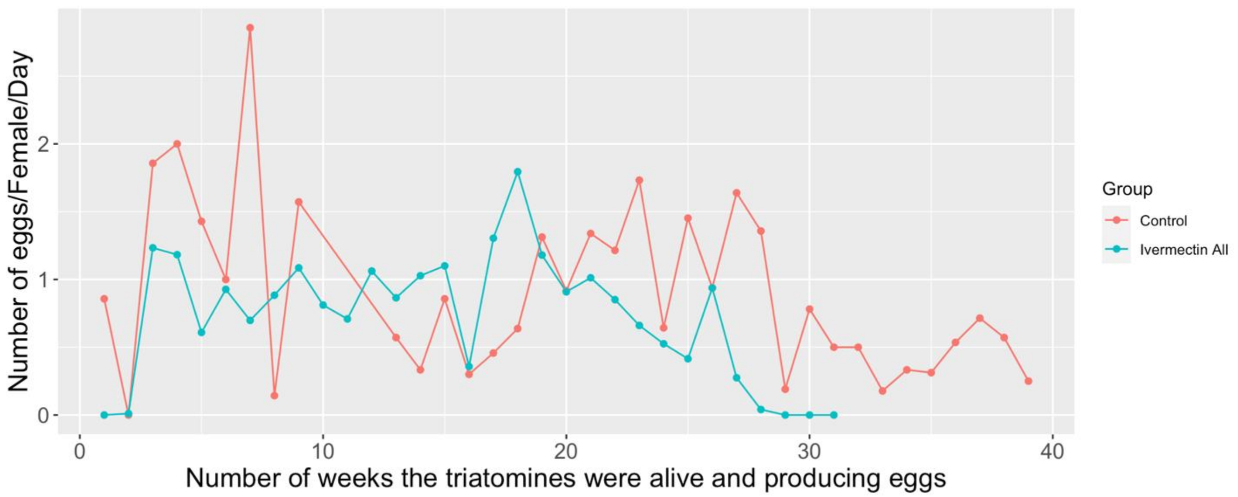

Figure S3: Number of eggs laid per female per day for each group (Control and Ivermectin; Ivermectin here represents the average eggs/female/day of days 7, 30, and 45) over time. For both groups, ‘week 1’ was the first instance a female was recorded in the group.

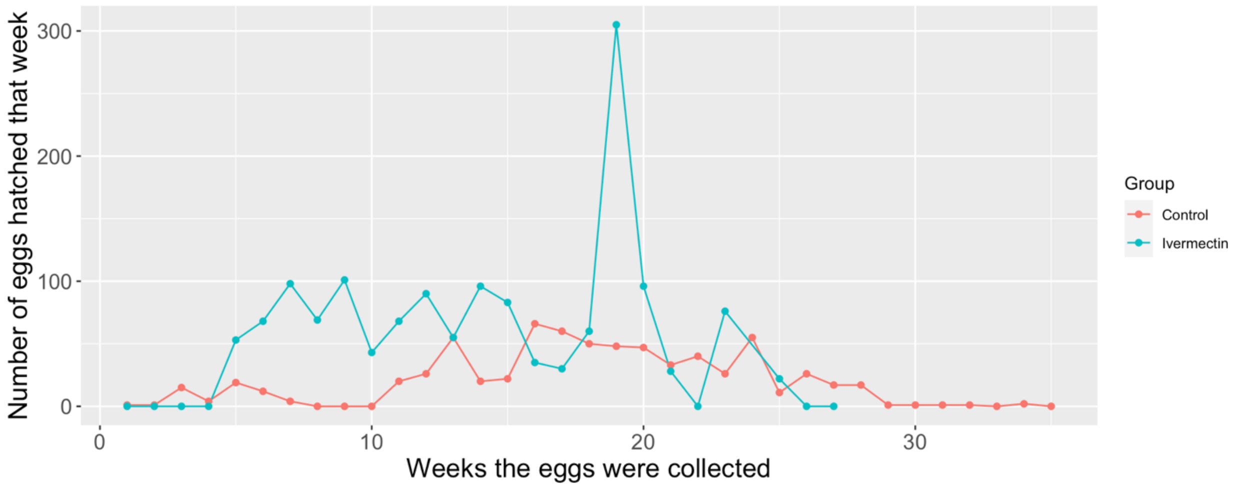

Figure S4: The raw number of eggs that hatched in control and ivermectin groups over time (Control and Ivermectin; Ivermectin here represents the total counts of the three days 7, 30, and 45 egg production and hatching). For both groups, ‘week 1’ was the first instance an egg hatched in the group.
